# Survey on Urinary Levels of Aflatoxins in Professionally Exposed Workers

**DOI:** 10.3390/toxins9040117

**Published:** 2017-03-24

**Authors:** Fulvio Ferri, Carlo Brera, Barbara De Santis, Giorgio Fedrizzi, Tiziana Bacci, Lorena Bedogni, Sauro Capanni, Giorgia Collini, Enrica Crespi, Francesca Debegnach, Patrizia Ferdenzi, Angelo Gargano, Daniela Gattei, Ferdinando Luberto, Ines Magnani, Massimo Giuseppe Magnani, Pamela Mancuso, Simonetta Menotta, Stefania Mozzanica, Milva Olmi, Giuseppe Ombrini, Orietta Sala, Sabina Soricelli, Massimo Vicentini, Paolo Giorgi Rossi

**Affiliations:** 1 Servizio Prevenzione Sicurezza Ambienti di Lavoro, SPSAL—AUSL, 42122 Reggio Emilia, Italy; fulvio.ferri@ausl.re.it (F.F.); lorena.bedogni@ausl.re.it (L.B.); sauro.capanni@ausl.re.it (S.C.); enrica.crespi@ausl.re.it (E.C.); patrizia.ferdenzi@ausl.re.it (P.F.); angelo.gargano@ausl.re.it (A.G.); daniela.gattei@ausl.re.it (D.G.); magnanines@gmail.com (I.M.); massimo.magnani@ausl.re.it (M.G.M.); stefania.mozzanica@ausl.it (S.M.); milva.olmi@ausl.re.it (M.O.); giuseppe.ombrini@ausl.re.it (G.O.); 2 Laboratorio Nazionale di Riferimento (LNR) per le Micotossine—Istituto Superiore di Sanità, 00161 Roma, Italy; carlo.brera@iss.it (C.B.); barbara.desantis@iss.it (B.D.S.); francesca.debegnach@iss.it (F.D.); sabina.soricelli@gmail.com (S.S.); 3 Istituto Zooprofilattico Sperimentale di Lombardia ed Emilia Romagna—Reparto Chimico, 40127 Bologna; giorgio.fedrizzi@izsler.it (G.F.); simonetta.menotta@izsler.it (S.M.); 4 Agenzia Regionale per la Prevenzione, l’Ambiente e l’Energia—ARPAE, 42122 Reggio Emilia, Italy; tbacci@arpae.it (T.B.); orietta2008@live.it (O.S.); 5 Servizio Interaziendale di Epidemiologia, AUSL, Reggio Emilia, 42122 Reggio Emilia, Italy; ferdinando.luberto@ausl.re.it (F.L.); pamela.mancuso@ausl.re.it (P.M.); massimo.vicentini@ausl.re.it (M.V.); paolo.giorgirossi@ausl.re.it (P.G.R.); 6 Arcispedale Santa Maria Nuova, IRCCS, 42123 Reggio Emilia, Italy

**Keywords:** aflatoxins, aflatoxin M_1_, animal feed, maize, occupational exposure

## Abstract

Feed mill workers may handle or process maize contaminated with aflatoxins (AFs). This condition may lead to an unacceptable intake of toxins deriving from occupational exposure. This study assessed the serological and urinary levels of AFs in workers exposed to potentially contaminated dusts in two mills. From March to April 2014, blood and urine samples were collected, on Monday and Friday morning of the same working week from 29 exposed workers and 30 non-exposed controls. AFs (M_1_, G_2_, G_1_, B_1_, B_2_) and aflatoxicol (AFOH) A were analyzed. Each subject filled in a questionnaire to evaluate potential food-borne exposures to mycotoxins. AFs contamination in environmental dust was measured in both plants. No serum sample was found to be positive. Seventy four percent of urine samples (73.7%) revealed AFM_1_ presence. AFM_1_ mean concentration was 0.035 and 0.027 ng/mL in exposed and non-exposed workers, respectively (*p* = 0.432); the concentration was slightly higher in Friday’s than in Monday’s samples, in exposed workers, 0.040 versus (vs.) 0.031 and non-exposed controls (0.030 vs. 0.024, *p* = 0.437). Environmental AFs contamination ranged from 7.2 to 125.4 µg/kg. The findings of this study reveal the presence of higher AFs concentration in exposed workers than in non-exposed controls, although these differences are to be considered consistent with random fluctuations.

## 1. Introduction

Aflatoxin B_1_ (AFB_1_) is a well-known carcinogenic mycotoxin classified by IARC in group 1 (carcinogenic agent to humans), that has shown a highly significant correlation with hepatocellular cancer incidence or mortality [1,2,3]. Due to the contamination of a considerably high amount of agricultural products worldwide (namely cereals and oil seeds), the main route of human exposure is via food ingestion, but other routes, such as skin contact and inhalation, in particular, have been found to play a role. While literature has placed great value on dietary exposure [4], the actual impact on health by exposure via skin contact and inhalation is not clear and data are not fully consistent [2,3,4,5]. Inhalation may be of particular concern in occupational exposure when associated with environmental conditions during the processing and handling of contaminated commodities. In fact, airborne concentration in some working places, such as feed or spice plant factories may be high [5].

The CAREX (CARcinogenEXposure) database provides selected exposure data and documented estimates of the number of exposed workers by country, carcinogen and industry [6]. From this database, the list of activities with a risk of aflatoxin exposure in Europe comprises among others, education services, research and scientific institutes, food manufacturing and veterinary services.

Several works, recently reviewed by Fromme et al. [7], reported a range of considerable levels of AFB_1_ in the air and in settled dusts of workplaces, highlighting a risk of exposure in special occupational settings. Thus, the risk of occupational exposure to aflatoxins has been assessed worldwide and Autrup [8,9] and Viegas [10,11,12] highlighted and reported evidence of exposure to AFB_1_ for workers in animal feed plants with or without comparison with non-exposed control groups. While exposure by inhalation has rarely been studied, absorption following oral exposure has been extensively explored and AFB_1_ showed to have a rapid uptake with 95% of kinetic urinary elimination occurring in 24 h [13]. After ingestion of contaminated food, AFB_1_ is metabolized by a number of cytochrome P450s, which generates two reactive epoxides (endo- and exo-8,9-epoxides), a number of hydroxymetabolites (aflatoxin M_1_, aflatoxin Q_1_, aflatoxin P_1_) and a reduction product (aflatoxicol, AFOH). The exo-epoxydes form a stable adduct with N7 moiety of guanine and this adduct, together with AFM_1_, has been observed in individuals exposed to AFB_1_ through their diet [14].

The measurement of the toxin and its products of biotransformation in humans have several problems both due to biological and analytical limits. Blood samples are difficult to handle and achieve and some of the standards for the analyses are not available (i.e., L7-Lysine and N7-Guanine); moreover, the tests are not harmonized among laboratories, being limited to research use and not to clinical use.

Extremely dry seasons increase the risk of the growth of aflatoxin-producing moulds in maize cultivars (*Zeamays*) [15]. In 2012, North Italy experienced an extremely dry season. Consequently, in the feed sector, an enormous amount of feedstuffs with high levels of AFB_1_ had to be managed by the feed companies which applied cleaning procedures and optical sorting by UV-ray techniques, in order to decrease contamination before the first-stage processing or use in the bio-energy sector [16]. Thus, during the 2012–2013 season, it was estimated that a large number of workers in the feedstuff sector in Northern Italy could presumably be exposed to contaminated maize dusts with unusually high levels of aflatoxins. The Service for Health and Prevention in Workplaces of the Local Health Unit (LHU) in Reggio Emilia started an investigation in order to assess the level of exposure to AFs of workers in two plants, one feed milling plant (Plant A) and a drying and sorting cereal plant (Plant B), both working on contaminated maize in the study period.

The objective of this study was to assess the level of aflatoxins in serum and urine samples of a group of workers. In the absence of lysine and guanine standards, analyses of AFB_1_, AFB_2_, AFG_1_, AFG_2_, AFM_1_, and AFOH were performed. The investigation also included analyses of AFs on environmental dusts collected from pumps and personal device filters that were placed both in plants and on workers. In addition, with the aim of obtaining information on the entire investigated worker group’s dietary habits, a food questionnaire was submitted.

## 2. Results

### 2.1. Dust Samples

Table 1 and Table 2 summarize the aflatoxin values found in filters (from airborne dust and personal devices, respectively). The pumps were placed only in areas where dust was present and on workers presumably exposed to dust. The analytical results of the airborne filters showed levels of aflatoxins in several areas of both plant A and plant B (Table 1), while personal air-filter devices showed aflatoxins only in plant A, but not in plant B, where only one personal air-filter was examined (Table 2) and only one duty with possible exposure has been tested. AFB_1_ was found in four out of seven areas of plant A: filters placed in production and unloading areas (area 1, 3, and 4) showed values in the range 1.2 μg/kg–7.2 μg/kg; AFB_1_ was found to be positive in IOM samplers (SKC, Eighty Four, PA 15330, USA) located in unloading and sorting areas of plant A. By expressing the values units as ng of aflatoxin per cubic meter of air, it resulted that the area at higher risk of exposure for workers was the unloading area in plant A (0.027 ng/m^3^). In plant B, despite having the highest values of AFB_1_ in dusts, no aflatoxin contamination was found from the personal samplers, most probably due to the low amount of total airborne dust collected. However, it must be considered that the workers commonly spend a few minutes per day in storage and sorting rooms, and the unloading and uploading operations are carried out on a closed shovel.

### 2.2. Serum and Urine Samples

Fifty-one out of 55 exposed workers from plant A were available for sampling on both days. Out of these, 27 workers were randomly selected. In plant B, all three exposed workers agreed to participate and were included. One worker of plant A provided only the Friday morning sample, because he was ill and was not replaced. Finally, data on 29 exposed workers were available.

The control group (*N* = 34) was selected from 152 operators of the LHU (district of Reggio Emilia) who showed their availability to participate. Twenty-one out of 34 workers were randomly selected. In addition, all nine white-collar workers employed in plant A agreed to participate as non-exposed controls; in total, data on 30 non-exposed controls were finally available.

The mean age was 50.2 years, ranging from 48.8 years for the exposed group and 51.5 years for non-exposed group. The mean weight was 81.5 kg, similar in the two groups (Table 3).

No quantifiable presence of free aflatoxins was found in serum samples (Limit of Detection, LoD = 0.025 ng/mL for AFB_1_, AFG_1_, AFM_1_ and AFOH and 0.006 ng/mL for AFB_2_ and AFG_2_). As for urine, AFB_1_, AFB_2_, AFG_1_, AFG_2_ and AFM_1_ quantifiable levels were found. None of the samples was positive for AFOH. More specifically, 87 (73.7%) out of 118 urine samples were positive for AFM_1_ (41 in exposed workers and 46 in non-exposed workers), 13 (11.1%) for AFG_2_ (five in exposed workers and eight in non-exposed workers), one (0.8%) for AFG_1_ (in exposed workers), one (0.8%) for AFB_2_ (in non-exposed workers), one (0.8%) for AFB_1_ (in non-exposed workers) (Table 4).

Considering the low number of positive results in urine for AFB_1_, AFB_2_, AFG_1_, AFG_2_ and AFOH, all the analyses and comparisons were conducted for the sole presence of AFM_1_ in urine. Table 4 summarizes the number and proportion of samples that were positive for AFM_1_, mean, confidence interval at 95% and median, merging the exposed and non-exposed worker groups.

The proportion of positive samples for AFM_1_ in exposed vs. non-exposed individuals was comparable, both considering Friday and Monday morning together [70.7%, 95% Confidential Interval (CI): 57.3%–81.9% vs. 76.7%; 95%CI: 64.0%–86.6%] and considering samples collected on Friday morning (62.1%, 95%CI: 42.3%–79.3% vs. 76.7%, 95%CI: 57.7%–90.1%) and on Monday morning (79.3%, 95%CI: 60.3%–92.0% vs. 76.7%, 95%CI: 57.7%–90.1%) (Table 5).

Table 5 summarizes both scenarios, divided by group and by day (Monday or Friday) for exposed and non-exposed workers in terms of number of positive samples, mean and median values. The mean concentration of AFM_1_ (including negative samples treated by a lower bound approach) was similar in the two groups in all samples (0.035 ng/mL in exposed workers and 0.027 ng/mL in non-exposed workers; *p* = 0.432). Samples taken on Friday in the exposed group had a slightly higher mean concentration than those taken on Monday morning; the difference may be due to random fluctuations (0.031 ng/mL and 0.040 ng/mL; *p* = 0.289). The difference between Monday and Friday samples was slightly stronger in the exposed group, but a similar effect was also observed in the non-exposed group, (0.009 ng/mL vs. 0.006 ng/mL; *p* = 0.437).

Six samples with values higher than mean plus 1.96 SD, i.e., higher than 0.141 ng/mL, belonging to the exposed group, were investigated separately. Within the exposed group, the highest AFM_1_ urine concentration was found on Friday (0.399 ng/mL), with the other three being intermediate concentration levels, taken on Friday (0.157 ng/mL both) and on Monday (0.161 ng/mL). Two other extreme values were obtained in samples from the non-exposed individuals: one sample taken on Monday (0.180 ng/mL), and one on Friday (0.259 ng/mL). Finally, there was no clear association between concentration of urine AFM_1_ and work duty or main working area (Table 6), even if the highest concentration was found in the unloading area of plant A.

## 3. Discussion

Although it is known that the main route of exposure to AFB_1_ is through diet, inhalation can constitute an additional route in occupational sites. This study, therefore, aimed to ascertain if a group of workers could be considered at risk because of exposure to contaminated dust in occupational environments.

Despite the treatment with protease and subsequent clean-up, it was not possible to detect free AFB_1_ in sera. Serum albumin adducts of AFB_1_ in blood are considered the main biomarker but because of the absence of AFB_1_-lysine standard, the method was unable to quantify the biomarker in sera samples. Thus, due to the unquantifiable presence of free aflatoxins in serum and the low number of positive results in urine for AFB_1_, AFB_2_, AFG_1_, AFG_2_ and AFOH, the study focused on AFM_1_ in the urine of a group of exposed and non-exposed workers. AFM_1_, considered one of the main recoverable urinary metabolites in humans [17], is a phase I hydroxyl-metabolite with a reduced toxicity, namely around 10% of AFB_1_.

Aflatoxin B_1_, AFB_2_, AFG_1_ and AFOH were found in less than 1% of the samples while AFG_2_ was found in 11% of the urine samples. While these small percentages for AFB_1_, AFB_2_, AFG_1_ and AFOH are in agreement with the route of excretion via urine, the value for AFG_2_ represents an unusual result for the urine sample.

AFM_1_ was found in the majority of the urine samples (>60%) in both groups. The mean level of AFM_1_ was comparable in exposed and non-exposed subjects and even the higher values were equally distributed in the two groups with the same extent. Given the relatively short half-life of aflatoxins, the Monday samples were supposed to have lower levels of aflatoxins attributable to work exposure than the corresponding Friday samples. However, only a slightly higher level was found in samples collected at the end of the working week than in the Monday morning sample, with large individual variability that make this difference largely compatible with random fluctuations (*p* = 0.29). We also found a slight increase in AFM_1_ concentration from Monday to Friday in the non-exposed group and the only plausible explanation for this change is a random fluctuation. Finally, the highest values of urinary AFM_1_ concentration were found both in exposed and non-exposed people, with prevalence, among the exposed workers who spent time in the unloading area, which actually proved to be the most contaminated area both for environmental and personal airborne dust samples.

To exclude the possible confounding components due to food contamination, we collected questionnaires about the consumption of foods at high risk of contamination. The only food that was associated with both exposure and AFM_1_ urine level was the chili powder. The analyses were replicated only for those who did not consume chili powder in the three days before sampling. The results of this sensitivity analysis suggest a slightly higher concentration of aflatoxins in the exposed workers compared to non-exposed controls (not significant, *p* = 0.07), but no increase in Friday compared to Monday among the exposed workers.

As a consequence, according to the present study design, no conclusive evidence for establishing a causal link between working exposure and serum or urine level of aflatoxins can be assumed, with the very small difference being attributable to random fluctuations or to background contamination level. However, the results highlight a general exposure to aflatoxin M_1_ as a consequence of AFB_1_ or AFM_1_ contaminated food and/or dairy products, respectively. In fact, in comparison to another work conducted in Italy [18], while the mean values are of the same magnitude (0.042 ng/mL vs. 0.07 ng/mL), the overall percentage (73.7%) of positives samples found in this study is much higher than in Solfrizzo’s study (6%) [18]. Few previous studies have measured the exposure of workers in different occupational sites. Most studies assessed the presence of aflatoxins in dust or air particulates, highlighting a possible risk of exposure. These studies showed that some industrial plants might reach AFB_1_ levels from 8 μg/kg [8,9] up to 5100 μg/kg in settled dust [19].

The environmental investigations in this study revealed a considerable concentration of dust in both plants, higher than the threshold considered by international agencies for worker safety [20,21,22]. On the other hand, the concentration of AFs in dust was low for plant A (maximum AFB_1_ 7.2 μg/kg), where the raw materials are checked before being unloaded, and quite high for plant B (maximum AFB_1_ 125.4 μg/kg), where highly contaminated maize is accepted for decontamination. The personal samplers, which should be considered more representative of the actual level of dust and contaminants inhaled by workers, were positive only in plant A (maximum AFB_1_ 5.6 μg/kg). Even if it was not possible to collect proper amounts of total airborne dusts, plant B is characterized by highly automated machinery, closed shovel and personal protection devices of the worker, that limit the exposure to very low level of dusts. Hence, the area with the highest risk of personal exposure to aflatoxins was the unloading area of plant A. It is interesting to note that AFB_1_ is, by far, the most frequently identified aflatoxin in the two plants and that AFG_1_ was never found.

Regarding the daily occupational exposure emerging from this study, values ranging from 0.0028 ng/m^3^ to 0.027 ng/m^3^ in plant A and from 0.120 ng/m^3^ to 1.505 ng/m^3^ in plant B were obtained, respectively. Considering personal samplers, in our setting, individual exposure to AFB_1_ ranged from 0.016 to 0.052 ng/m^3^, which is lower than most of the values observed in previous studies, where the maximum values ranged from 0.4 to 7.6 ng/m^3^ (shelling plant) [23], or from 124 ng/m^3^ to 4849 ng/m^3^ (bin cleaning) [19].

Only one study analysed individual exposure with biomarkers. The study compared the serum albumin adduct levels in workers after a holiday period and after 4 weeks of work [9] immediately after the holidays. Only two workers out of 45 showed AFB-albumin adduct presence, while seven workers out of 45 showed the presence of albumin-adducts after the working period. Unfortunately, the analytical methods used in ours and the Danish study are different and do not allow a direct comparison to be made. Furthermore, it must be considered that the half-life of AFB_1_ in blood is about 90 h [24], while the wash out period in our study was about 62 h (from Friday 6 p.m. to Monday 8 a.m.). Consequently, the baseline level of Monday morning could contain more than half of the aflatoxins accumulated during the working week; on the contrary, in the Danish study, the wash out period was longer and probably allowed complete decontamination.

The environmental analyses and results of the urine samples suggest that in plants treating raw materials with a low level of contamination (i.e., 20 µg/kg for swine and 4 µg/kg for milk cattle), the exposure of workers can be reduced to levels similar to those of the general population by controlling dust levels. In fact, if in plant A the level of environmental dust was below the internationally recommended thresholds (for the moment not established by law in Italy), the level of individual contamination would be very low. In situations with higher contamination of raw materials, as in plant B, high automation and closed shovel, restricting the personal exposure of workers, were revealed to be effective in preventing detectable contamination of inhaled powders.

### Strengths and Limits

The sampling methods were strictly controlled and identical in workers and controls. The study design has an internal and external control, which should give strong validity to any possible association between AFM_1_ levels and work exposure; furthermore, the analytical technique used for aflatoxin detection provided good LoD values. On the other hand, it is still possible to scrutinize the serum samples for AFB_1_-albumin adduct in order to reliably assess long-term exposure to AFs.

The results and how they can be interpreted should be discussed in the perspective of previous studies and of the working hypotheses. The findings and their implications should be discussed in the broadest context possible. Future research directions may also be highlighted.

## 4. Conclusions

The results of the study suggest higher AF concentration in exposed workers in the two investigated mills treating highly contaminated maize than in non-exposed controls, although these differences are to be considered consistent with random fluctuations.

The aflatoxin intake by the exposed workers led to a maximum urine AF concentration similar to that observed with the control group, where contamination could only be derived from the food-borne exposure.

## 5. Materials and Methods

### 5.1. Setting

The study was conducted under the supervision of the Local Health Unit and was approved by the Ethical Committee of the Reggio Emilia province (act number 2013/DS/0086 30/12/2013).

The investigation was conducted in two plants located in the same province, in Northern Italy: plant A, a large feedstuff plant producing, every year, about 540,000 metric tons of feedstuffs (e.g., flour, compost and pellet), nearly 100,000 metric tons derived from maize; and plant B, a small drying and sorting cereal plant producing nearly 27,000 metric tons of cereal, mainly maize (18,000 metric tons).

The Plant Management of the company agreed to participate according to the criteria and principles set by Italian legislation on workers’ health and safety and the study on human samples was also agreed with trade union representatives and the competent medical team. The workers were informed about the purpose of the study through a public meeting. During the meeting, formal consent for participation was individually requested and signed.

### 5.2. Study Design

Dust samples were collected by the LHU officers with gravimetric samplers located in the main areas where cereals were handled (five areas in plant A and 1 area in plant B). Blood and urine samples were collected twice for each individual: on Monday morning before starting work on the first working day of the week, and on Friday morning, at the end of the working week.

### 5.3. Samples

Dust samples were collected using two types of pumps: static pumps Zambelli (ZP1: flow 20 L/min, glass micro-fibre filters 47 mm diameter) for inhalable powder placed in fixed positions; and high volume air sampler pumps (Tish Environmental, Inc., Mod.TE-5170-BL: flow 1400 L/min, glass fibre filter 254 mm × 203 mm). Pumps were only set in areas where dust was present; no control samples from clean areas were placed.

To assess possible individual exposure by inhalation, IOM samplers for inhalable dusts were used (25 mm diameter micro fibre filters), connected with a Kronos ZS05S (Zambelli srl, 20010 Bareggio, Milano, Italy) pump and Airchek XR5000 (SKC) pump (flow 2 L/min). The individual samplers were applied to 18 workers enrolled in the production line 7 in the unloading line of plant A and one worker on board a mechanical shovel of plant B, over three working days.

To guarantee a sufficient amount of sample in a short time frame, a number of filters from various pumps were assembled in the same area and personal samplers from various subjects involved in the same activity were pooled and the particulates were analysed together. The planned sample included 120 biological samples from 60 male individuals: Monday and Friday samples from 30 exposed workers and 30 non-exposed workers as controls. The exposed workers group was formed by selecting individuals from the technical staff, namely, 27 workers from plant A and three from plant B; as for the non-exposed control group, nine individuals, who had no contact with contaminated airborne areas, were from plant A and 21 were LHU employees.

The human biological samples and dust environmental samples were collected from 31 March 2014 to 18 April 2014.

The collection of human specimens was carried out on the work site under a protocol pre-approved by a physician and a nurse. The urine of exposed workers was collected in the morning, after the night’s rest and delivered to the medical staff before starting the morning shift. At this time, a blood sample was taken and a questionnaire about eating habits and duties carried out the previous week or the days before (Monday’s or Friday’s withdrawal) was administrated. The collection of samples and the administration of questionnaires was conducted for two consecutive weeks to investigate all enrolled workers, at the beginning of the morning shift.

The biological samples and questionnaires of the control group were collected in the first week. Blood samples were collected in 10 mL cryogenic tubes and immediately transported in refrigerated boxes at 0 °C to the local reference laboratory. The blood samples were left for 2 h at room temperature to allow for coagulation, and then centrifuged. At least 5 mL of serum was stored and then dispatched under controlled temperature (−20 °C) to the National Reference Laboratory (NRL) for mycotoxins in Rome (Istituto Superiore di Sanità). Urine samples were collected using sterile tubes VACUETTE^®^ Urine System, 10 mL (Greiner Bio-One Gmbh, 4550 Kremsmüster, Austria). Serum and urine, stored at −20°C, were sent together to the NRL.

### 5.4. Food Questionnaire

In order to gain information on possible food-borne exposure to mycotoxins, a questionnaire on the food eaten during the week preceding the biological fluid collection was administrated to the participants. The requested information focused on food products that are a potential source of aflatoxin contamination (i.e., corn and other cereals, rice, spices, dried fruits, dehydrated fruits, dairy products, fresh milk, fresh cheese and hard cheese). Furthermore, some other foods, known to be not at risk for aflatoxin exposure, were also included in the questionnaire (i.e., fish, molluscs or crustaceans, fresh meat, fresh fruit). For each food item, participants were asked to specify the amount and frequency of what they ingested during the week preceding the sample collection. The questionnaire also collected information on work duties, age and weight.

### 5.5. Laboratory Analyses

#### 5.5.1. Analysis of Dusts

The sample powder was mixed with methanol (JT Baker Center Valley PA USA) solution (CH_3_OH 80%), then homogenized (mixing shaker IKA Werke Staufen Germany) and centrifuged (J-HC centrifuge Beckman Coulter USA). The supernatant was dissolved in PBS (all reagents were from Sigma Aldrich Saint Luis MO USA) and cleaned up by an IAC (Aflastar, ROMER Labs DH Erber Campus Getzersdorf Austria). The IAC was eluted with 2 mL of methanol acetic acid solution (JT Baker Center Valley PA USA) (CH_3_OH: 2% CH_3_COOH). The total amount of 2 mL was analysed in LC-MS/MS (Liquid chromatography–mass spectrometry) (Waters corporation, Milford, MA, USA) with a binary gradient of water and acetonitrile with 0.2% formic acid in a reverse-phase high Performance LC column (Luna C18, 5 µ, 2.1 mm × 150 mm; Phenomenex inc., Torrance, CA, USA). Fragmentation was performed in positive ionization. The quantification was on mass/charge 241.0 for AFB_1_, 243.0 for AFB_2_, 200.0 for AFG_1_ and 188.9 for AFG_2_. The sensitivity of the method was 0.5 µg/kg for each aflatoxin. The analytical method was previously validated for food matrices and applied to dust samples.

#### 5.5.2. Analysis of Serum and Urine

Serum and urine samples were analysed for AFB_1_, AFB_2_, AFG_1_, AFG_2_, AFM_1_ and AFOH. After the assignment of a numerical identification code for guaranteeing traceability, blind analyses were carried out on serum and urine samples (summing up Monday and Friday exposed and non-exposed workers).

An HPLC-FLD method, previously set and validated in-house for AFs, was used for the six mycotoxins in serum and urine. Mycotoxins in serum samples were diluted with 400 μL of phosphate buffer saline (PBS), and cleaned up using a specific immunoaffinity column (IAC, AFLAPREP, R-Biopharm Rhone, Glasgow, UK).

Extraction from urine was carried out by diluting the samples with PBS, followed by the same clean-up step as in serum samples. A reverse-phase HPLC-FLD method of analysis, under isocratic conditions (mobile phase: MeOH:H_2_O:AcCN, 20:20:60 v/v; flow rate: 1.2 mL/min; Column Phenomenex Luna C18, 5 µ, 2.1 mm × 150 mm) was used. A post-column electrochemical derivatisation step was used by connecting in linean electrochemical cell (Coring-cell, Coring System Diagnostix GmbH, Germany), and adding to the mobile phase the derivatizing agent (100 μL HNO_3_ 65% and 119 mg KBr in the mobile phase). The fluorimetric detection was obtained setting appropriate excitation (λex) and emission (λem) wavelengths (AFs, λex/λem 365/440 nm, *t* = 0–17 min; AFOH, λex/λem 333/418 nm, *t* = 17–25 min).

The validation parameters such as relative standard deviation of repeatability, recovery and limit of detection (LoD) are reported in Table 7. Trueness was tested by performing replicate analyses (*n* = 8) both in serum and in the urine of spiked samples at contamination levels of 0.500 ng/mL for AFB_1_ and AFG_1_; 0.250 ng/mL for AFM_1_ and AFOH; 0.125 ng/mL for AFB_2_ and AFG_2_ in serum; and 0.500 ng/mL for AFB_1_, AFG_1_, AFM_1_ and AFOH; and 0.125 ng/mL for AFB_2_ and AFG_2_ in urine.

### 5.6. Data Analysis

In this study, the percentage of positive urine samples, mean with relative 95% confidence interval (95%CI) and median values of concentration were reported, by group (exposed and non-exposed) and by day (Monday and Friday). The left-censored values were reported adopting a lower-bound approach. Two formal tests of hypothesis were postulated: in the first comparison, the null hypothesis was that the difference between mean concentration in all samples in the exposed and in the control group was zero; in the second, the null hypothesis was that the difference between Friday and Monday samples in the exposed and in the control group was equal. The hypotheses were tested using t-student distribution with alpha 0.05, after checking for normal distribution of the differences with Shapiro–Wilk’s test.

In order to assess whether extreme values were associated with exposure, the exposure status of the samples over 1.96 standard deviation (SD) is reported in detail. In order to exclude confounding values/factors due to exposure to aflatoxins via diet, the distribution of food at high and medium risk of aflatoxin contamination in the two groups was compared by a bivariate analysis, where any association with χ^2^ *p*-value < 0.1 was considered as a possible confounder. In addition, a descriptive analysis was performed reporting the mean level of AFM_1_ among the exposed subjects by activity, grouped according to exposure risk.

## Figures and Tables

**Table 1:** Concentration values in airborne dust (μg/kg) and in air particulates (ng/m^3^) from pumps (static and high flow rate).

Pump Placement	Measurement Unit/Value	Plant A	Plant A	Plant A	Plant A	Plant A	Plant A	Plant A	Plant B	Plant B	Plant B	Plant B	Plant B
		Production area	Unloading area 1	Unloading area 2	Unloading area 3	Unloading area 4	Unloading Area 5	Integration and medication area	Storage	Sorting area 1	Sorting area 2	Sorting area 3	Sorting area 4
**Pump type**		Static (Zambelli)	Static (Zambelli)	High flow rate	High flow rate	High flow rate	High flow rate	High flow rate	Static (Zambelli)	Static (Zambelli)	High flow rate	High flow rate	High flow rate
**Assembled Filters**	***N* (Number of)**	14	6	1	1	1	1	1	3	3	1	1	1
**Total airborne dust**	**(mg)**	287	158	991	12916	904	524	306	247	157	1386	1568	1204
**Total air**	**m^3^**	113	42	703	579	557	466	348	21	22	470	462	491
**Dust conc mg/m^3^**	**Mean**	2.5	3.7	1.4	22.3	1.6	1.1	0.9	12.0	7.2	2.9	3.4	2.5
	**(range)**	(0.5; 5.2)	(1.7; 6.8)	-	-	-	-	-	(9.4; 14.5)	(5.2; 8.8)	-	-	-
**AFs conc**													
**AFB_1_**	**µg/kg powder**	1.9	7.2	-	1.2	1.7	-	-	125.4	41.3	72	55.1	49.1
	**(ng/m^3^ air)**	(0.005)	(0.027)		(0.027)	(0.003)			(1.505)	(0.298)	(0.212)	(0.187)	(0.120)
**AFB_2_**	**µg/kg powder**	0.5	1.9	-	-	-	-	-	6.90	3.3	4	3.2	2.8
	**(ng/m^3^ air)**	(0.001)	(0.007)						(0.083)	(0.024)	(0.012)	(0.009)	(0.008)
**AFG_1_**	**µg/kg powder**	-	1.1	-	-	0.7	-	-	7.60	1.4	5.1	4	3.3
	**(ng/m^3^ air)**		(0.004)			(0.001)			(0.091)	(0.010)	(0.015)	(0.012)	(0.010)
**AFG_2_**	**µg/kg powder (ng/m^3^ air)**	-	-	-	-	-	-	-	-	-	-	-	-
**Sum AFs**	**µg/kg powder**	2.4	10.2	-	1.2	2.4	-	-	139.9	46.0	81.1	62.3	55.2
	**(ng/m^3^ air)**	(0.006)	(0.038)		(0.027)	(0.004)			(1.679)	(0.332)	(0.239)	(0.208)	(0.138)

**Table 2:** Aflatoxin concentration values in airborne dust (μg/kg), and in air particulates (ng/m^3^) from personal samplers (IOM).

Pump Placement	Measurement Unit/Value	Investigated productive areas		
		Plant A Production Area	Plant A Unloading	Plant B Sorting
**Pump Type**		Personal sampler (IOM)	Personal sampler (IOM)	Personal sampler (IOM)
**Assembled Filters**	***N***	18	7	3
**Total Airborne Dust Collected**	**(mg)**	34.5	35.7	2.5
**Total Air (m^3^) Filtered**	**m^3^**	8.4	3.9	1.4
**Dust Concentration mg/m^3^**	**Mean (Range)**	3.9 (0.7–10.5)	9.4 (1.3–25.6)	1.8 (mechanical shovel)
**AF Concentration**				
**AFB_1_**	**µg/kg Powder (ng/m^3^ air)**	4.0 (0.016)	5.6 (0.052)	-
**AFB_2_**	**µg/kg Powder (ng/m^3^ Air)**	-	3.8 (0.030)	-
**AFG_1_**	**µg/kg Powder (ng/m^3^ Air)**	-	0.6 (0.010)	-
**AFG_2_**	**µg/kg Powder (ng/m^3^ Air)**	-	-	-
**Total AFs**	**µg/kg Powder (ng/m^3^ Air)**	4.0 (0.016)	10.0 (0.092)	-

**Table 3:** Distribution of workers by group, age and weight.

Groups	*N* Workers Plant A	*N* Workers Plant B	*N* Operators LHU	Total Volunteers	Mean Age (Range)	Mean Weight (Range)
Exposed	26	3	0	29	48.8 (34–67)	81.5 (63–100)
Non-Exposed	9	0	21	30	51.5 (28–64)	81.4 (60–104)
Total	35	3	21	59	50.2 (28–67)	81.5 (60–104)

**Table 4:** Number of positive samples, mean, median, 90° percentile values for all mycotoxins urine samples (-: not available).

Urine samples results	AFM_1_	AFG_2_	AFG_1_	AFB_2_	AFB_1_	AFOH
*N* of positives (%)	87 (73.7)	13 (11.1)	1 (0.8)	1 (0.8)	1 (0.8)	0
Mean (ng/mL)	0.042	0.057	0.058	0.007	0.010	-
Median (ng/mL)	0.017	0.019	0.058	0.007	0.010	-
90° percentile (ng/mL)	0.099	0.175	0.058	0.007	0.010	-

**Table 5:** Mean, median, number and proportion of urine samples (including negative samples) for AFM_1_ by group and by day.

Groups and days	*N* (%) of Positive Samples	Mean ng/mL	(95%CI)	Range (ng/mL)	Median (ng/mL)	Interquartile Range (ng/mL)
**Exposed Workers**						
Monday	23/29 (79.3)	0.031	(0.015–0.046)	(0–0.161)	0.009	0.052
Friday	18/29 (62.1)	0.040	(0.008–0.071)	(0–0.399)	0.006	0.030
Total	41/58 (70.7)	0.035	(0.018–0.052)	(0–0.399)	0.008	0.051
**Non-Exposed Controls**						
Monday	23/30 (76.7)	0.024	(0.008–0.039)	(0–0.180)	0.008	0.013
Friday	23/30 (76.7)	0.030	(0.010–0.049)	(0–0.259)	0.014	0.032
Total	46/60 (76.7)	0.027	(0.015–0.039)	(0–0.259)	0.009	0.025

**Table 6:** Level of urinary AFM_1_ by work duty and main working area.

Plant	Main Working Area	Unit/Task	*N* of Workers	Mean (ng/mL)	Min	Max
Plant A	All areas	Various	2	0.099	0.000	0.157
Plant A	Unloading	Receipt and upload raw materials	3	0.090	0.000	0.399
Plant A	Production	Pellet	1	0.045	0.006	0.084
Plant A	Production	Electric Control panel	3	0.042	0.000	0.161
Plant A	Production	Driver in bulk foodstuff	4	0.030	0.000	0.091
Plant B	Sorting	Drying and sorting	3	0.029	0.000	0.113
Plant A	Production	Packaging	1	0.027	0.015	0.040
Plant A	Production/Integrator and medication	Pellet/adding integrators and medications	1	0.026	0.000	0.051
Plant A	Production	Clearing/movement	1	0.018	0.010	0.025
Plant A	All areas	Maintenance	3	0.016	0.000	0.089
Plant A	Integrator and medication	Adding integrators and medications	4	0.011	0.000	0.057
Plant A	Production	Charging in bulk foodstuff	1	0.010	0.006	0.014
Plant A	Production	Charging packed foodstuff	1	0.005	0.000	0.009
Plant A	Production	Laboratory technician	1	0.000	0.000	0.000

**Table 7:** Validation performance parameters obtained for serum and urine.

Serum and urine parameters	AFB_1_	AFG_1_	AFB_2_	AFG_2_	AFM_1_	AFOH
**(a)**						
**Mean (ng/mL serum)**	0.555	0.499	0.105	0.555	0.251	0.161
**LoD ^a^ (ng/mL serum)**	0.025	0.025	0.006	0.006	0.025	0.025
**RSDr ^b^ (ng/mL serum)**	17.5	8.5	15.0	19.0	12.1	18.0
**Rec ^c^ (%)**	111	100	84	90	101	60
**(b)**						
**Mean (ng/mL urine)**	0.346	0.298	0.075	0.060	0.305	0.191
**LoD ^a^(ng/mL urine)**	0.010	0.006	0.006	0.004	0.002	0.025
**RSDr ^b^ (ng/mL urine)**	5.8	11.3	9.9	10.4	7.0	9.9
**Rec ^c^ (%)**	81	79	77	71	83	60

**^a^** LoD = Limit of Detection; ^b^ RSDr = Relative Standard Deviation repeatability; **^c^** Rec = Recovery factor.
